# Characteristics of imported *Plasmodium ovale* spp. and *Plasmodium malariae* in Hubei Province, China, 2014–2018

**DOI:** 10.1186/s12936-020-03337-y

**Published:** 2020-07-22

**Authors:** Jing Xia, Dongni Wu, Lingcong Sun, Hong Zhu, Kaijie Li, Juan Zhang, Wen Lin, Lun Wan, Huaxun Zhang, Si Liu

**Affiliations:** Institute of Parasitic Disease Control, Hubei Provincial Center for Disease Control and Prevention, Wuhan, 430079 China

**Keywords:** *Plasmodium ovale* spp., *Plasmodium malariae*, Importation, Latency periods, Misdiagnosis, Hubei Province, China

## Abstract

**Background:**

There have been an increasing number of imported cases of malaria in Hubei Province in recent years. In particular, the number of cases of *Plasmodium ovale* spp. and *Plasmodium malariae* significantly increased, which resulted in increased risks during the malaria elimination phase. The purpose of this study was to acquire a better understanding of the epidemiological characteristics of *P. ovale* spp. and *P. malariae* imported to Hubei Province, China, so as to improve case management.

**Methods:**

Data on all malaria cases from January 2014 to December 2018 in Hubei Province were extracted from the China national diseases surveillance information system (CNDSIS). This descriptive study was conducted to analyse the prevalence trends, latency periods, interval from onset of illness to diagnosis, and misdiagnosis of cases of *P. ovale* spp. and *P. malariae* malaria.

**Results:**

During this period, 634 imported malaria cases were reported, of which 87 *P. ovale* spp. (61 *P. ovale curtisi* and 26 *P. ovale wallikeri*) and 18 *P. malariae* cases were confirmed. The latency periods of *P. ovale* spp., *P. malariae*, *Plasmodium vivax*, and *Plasmodium falciparum* differed significantly, whereas those of *P. ovale curtisi* and *P. ovale wallikeri* were no significant difference. The proportion of correct diagnosis of *P. ovale* spp. and *P. malariae* malaria cases were 48.3% and 44.4%, respectively, in the hospital or lower-level Centers for Disease Control and Prevention (CDC). In the Provincial Reference Laboratory, the sensitivity of microscopy and rapid diagnostic tests was 94.3% and 70.1%, respectively, for detecting *P. ovale* spp., and 88.9% and 38.9%, respectively, for detecting *P. malariae*. Overall, 97.7% (85/87) of *P. ovale* spp. cases and 94.4% (17/18) of *P. malariae* cases originated from Africa.

**Conclusion:**

The increase in the number of imported *P. ovale* spp. and *P. malariae* cases, long latency periods, and misdiagnosis pose a challenge to this region. Therefore, more attention should be paid to surveillance of imported cases of *P. ovale* spp. and *P. malariae* infection to reduce the burden of public health and potential risk of malaria.

## Background

Malaria is one of the major global public health threats and causes substantial morbidity, mortality, and human suffering. In 2017, an estimated 219 million malaria cases occurred worldwide, with 435,000 deaths reported [1]. In humans, malaria is caused by five *Plasmodium* species (*Plasmodium falciparum*, *Plasmodium vivax*, *Plasmodium malariae*, *Plasmodium ovale* spp., and *Plasmodium knowlesi*) [2–5]. However, less emphasis is placed on malaria caused by *P. ovale* spp. and *P. malariae* compared with *P. falciparum* and *P. vivax*, since malaria caused by *P. ovale* spp. and *P. malariae* has a more benign clinical course than that caused by *P. falciparum*. Therefore, malaria caused by *P. ovale* spp. and *P. malariae* have been neglected and are understudied [6–8].

In recent years, the number of imported *P. ovale* spp. and *P. malariae* malaria cases has increased yearly in China [9, 10]. However, no local *P. ovale* spp. malaria cases have ever been reported in Hubei Province, and no local *P. malariae* malaria cases have been detected since 1963 [11]. Since 2013, no indigenous malaria cases have been reported in Hubei Province; furthermore, imported malaria cases, particularly those of *P. ovale* spp. and *P. malariae*, significantly increased, which resulted in increased risks during the malaria elimination phase [12, 13]. Patients infected with *P. ovale* spp. and *P. malariae* often had longer latency periods than those infected with *P. falciparum*, and they may present with malaria symptoms months or even years after returning from an endemic region [14, 15]. Therefore, accurate and prompt identification of malaria species is required for suitable use of anti-malarial drugs to reduce the burden of public health and potential risk of malaria.

In recent years, two sympatric species of *P. ovale* spp. have been distinguished: *P. ovale curtisi* and *P. ovale wallikeri* [5]. The morphology of these two sympatric species of *P. ovale* spp. was identical on microscopy, but the two can be differentiated by genetic typing [16, 17]. To date, however, there have been few epidemiological studies conducted of *P. ovale* spp. in Hubei. In light of the epidemiology of *P. ovale* spp. and *P. malariae*, a comprehensive analysis was required to explore the changing epidemiology and challenges encountered in Hubei with respect to malaria elimination. Thus, in this study, the data were obtained from the China National Disease Surveillance Information System (CNDSIS) during 2014–2018. This descriptive study was conducted to analyse the prevalence trends, latency periods, interval from onset of illness to diagnosis, and misdiagnosis of cases of *P. ovale* spp. and *P. malariae* malaria. The evidence of this study will help in providing a reference for preventing and managing imported *P. ovale* spp. and *P. malariae* malaria cases in Hubei Province.

## Methods

### Data collection

Data on malaria cases in Hubei Province from 2014 to 2018 were obtained from (CNDSIS). The following details of patients with malaria were extracted: gender, age, occupation, admission dates, symptom onset time, time of diagnosis, laboratory-confirmed results, travel history.

### Laboratory examinations

All cases were initially diagnosed using thick and thin blood film microscopy at hospitals or low-level Center for Disease Control and Prevention (CDC). Thereafter, all results were then sent to the Hubei Provincial Reference Laboratory for Malaria Diagnosis. All cases were confirmed by the Provincial Reference Laboratory for using thick and thin blood film microscopy, malaria rapid diagnostic tests (RDTs), and nested polymerase chain reaction (nested PCR). Thick and thin blood films were stained using a standard technique with Giemsa solution for 35 min; they were then examined by trained microscopists to diagnose and identify malaria species. Next, 5 μl of whole blood of each patient was analysed using the Ag Pf/Pan RDTs (Guangzhou Wondfo Biotech Co., Ltd., Guangzhou, China) following the manufacturer’s protocol. As nested PCR is more sensitive and specific in determining the malaria parasite species [18, 19]; for detecting the *Plasmodium* species and two sympatric subspecies of *P. ovale* spp. at the molecular level, nested PCR was performed by amplifying the 18SSU rRNA gene of the malaria parasite. The nested PCR protocol was followed: DNA isolation was extracted by using the QIAamp DNA Mini Kit (QIAGEN^®^) from the blood sample following the protocol. The first reaction of DNA amplification was performed by using rPLU5 and rPLU6 primers (Thermos^®^) [20]. The PCR solution had a total volume of 20 μl, comprising 3 μl of DNA solution, 0.2 μl of Taq DNA polymerase (Takara^®^), 1.1 μl of each primers (10 μmol/l), 1.6 μl of dNTPs (2.5 mmol/l), 4.0 μl of 5× Buffer and 9.0 μl of ddH_2_O. For the second reaction, there are five pairs of primers were used: rVIV1/rVIV2 (*P. vivax*), rFAL1/rFAL2 (*P. falciparum*), rMAL1/rMAL2 (*P. malariae*), rOVA1/rOVA2 (*P. ovale curtisi*) and rOVA1v/rOVA2v (*P. ovale wallikeri*). These were used to determine the *Plasmodium* species [17, 20]. The PCR solution had a total volume of 20 μl, comprising 2 μl of PCR product of the first reaction as the template, 10 μl of 2× DreamTaq PCR Master Mix (Fermentas^®^), 1.0 μl of each primers (10 μmol/l), add enough ddH_2_O to make up the volume to 20 μl. The incubation conditions of the two PCR rounds were as follows: 3 min at 94 °C; followed by 35 cycles of 30 s at 94 °C, 30 s at 58 °C, 1 min at 72 °C, and a final extension for 5 min at 72 °C. The PCR products of the second reaction were run on standard 1.5% agarose gels.

### Statistical analysis

Statistical analyses were performed using the SPSS software version 16.0 (SPSS Inc., Chicago, IL, USA). Owing to the lack of the exact date of malaria infection, the date of arrival in China was used as the date of malaria infection. The latency period, in days, was calculated for each case of malaria by subtracting the date of arrival in China from the date of symptom onset. The confirmed diagnosis interval in days was calculated from the onset of symptoms to laboratory confirmation in provincial CDC. The latency period and confirmed diagnosis interval were calculated and compared for all malaria cases except mixed *Plasmodium* infections. Because the latency periods and interval from the onset of illness to diagnosis had non-normal distribution, the differences were compared using Kruskal–Wallis *H* tests and two independent sample Mann–Whitney U tests, with p-values < 0.05 considered statistically significant. The ArcGIS software version 10.0 (ESRI Inc., Redlands, CA, USA) was used to produce figures of the geographic distribution of the countries of origin of the imported *P. ovale* spp. and *P. malariae* cases.

## Results

### Prevalence of imported *P. ovale* spp. and *P. malariae*

During 2014–2018, a total of 634 imported malaria cases were reported in Hubei Province (Table 1). *P. falciparum* (440), *P. vivax* (84), *P. ovale* spp. (87), *P. malariae* (18) and mixed infection (3) accounted for 69.4%, 13.2%, 13.7%, 2.8%, and 0.5% of all reported cases, respectively. The *P. ovale* spp. incidence ranged from 11 to 24 cases per year and that of *P. malariae* from 0 to 7 cases per year for the period 2014–2018. Among the 87 patients infected *P. ovale* spp. and 18 patients infected with *P. malariae*, there was only one patient infected with *P. ovale* spp., who was a Nigerian, the rest were all Chinese who worked in a malaria-endemic countries as migrant workers.

**Table 1:** Imported malaria cases in Hubei Province, 2014–2018

Year	All cases	*P. falciparum* N (%)	*P. vivax* N (%)	*P. ovale* N (%)	*P. malariae* N (%)	Mixed infection N (%)
2014	140	105 (75.0)	21 (15.0)	11 (7.9)	3 (2.1)	0 (0)
2015	120	91 (75.8)	9 (7.5)	14 (11.7)	5 (4.2)	1 (0.8)
2016	151	103 (68.2)	21 (13.9)	22 (14.6)	3 (2.0)	2 (1.3)
2017	96	49 (51.0)	16 (16.7)	24 (25.0)	7 (7.3)	0 (0)
2018	127	94 (74.0)	17 (13.4)	16 (12.6)	0 (0)	0 (0)
Total	634	440 (69.4)	84 (13.2)	87 (13.7)	18 (2.8)	3 (0.5)

**Fig. 1:**
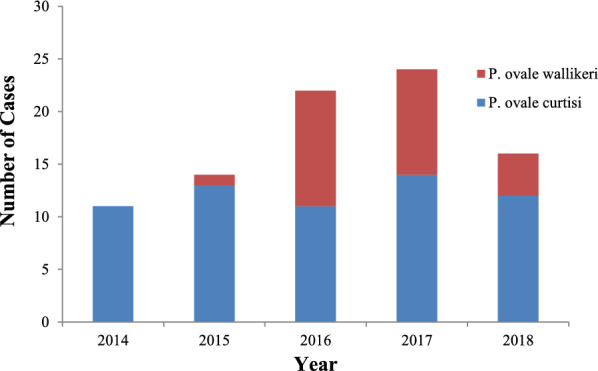
Number of *P. ovale curtisi* and *P. ovale wallikeri* malaria cases in Hubei Province, 2014–2018

For all 86 Chinese *P. ovale* spp. patients, the median and interquartile range of the duration of stay overseas was 345 days (167–523). For all *P. malariae* patients, the median and interquartile range of duration of stay overseas was 360.5 days (222.5–667.8). In total, 87 *P. ovale* spp. cases, including 61 *P. ovale curtisi* and 26 *P. ovale wallikeri* (the first *P. ovale wallikeri* case was discovered in 2015) (Fig. 1), and 18 *P. malariae* cases were confirmed by nested PCR.

### Latency period between arrival in China and illness onset

**Fig. 2:**
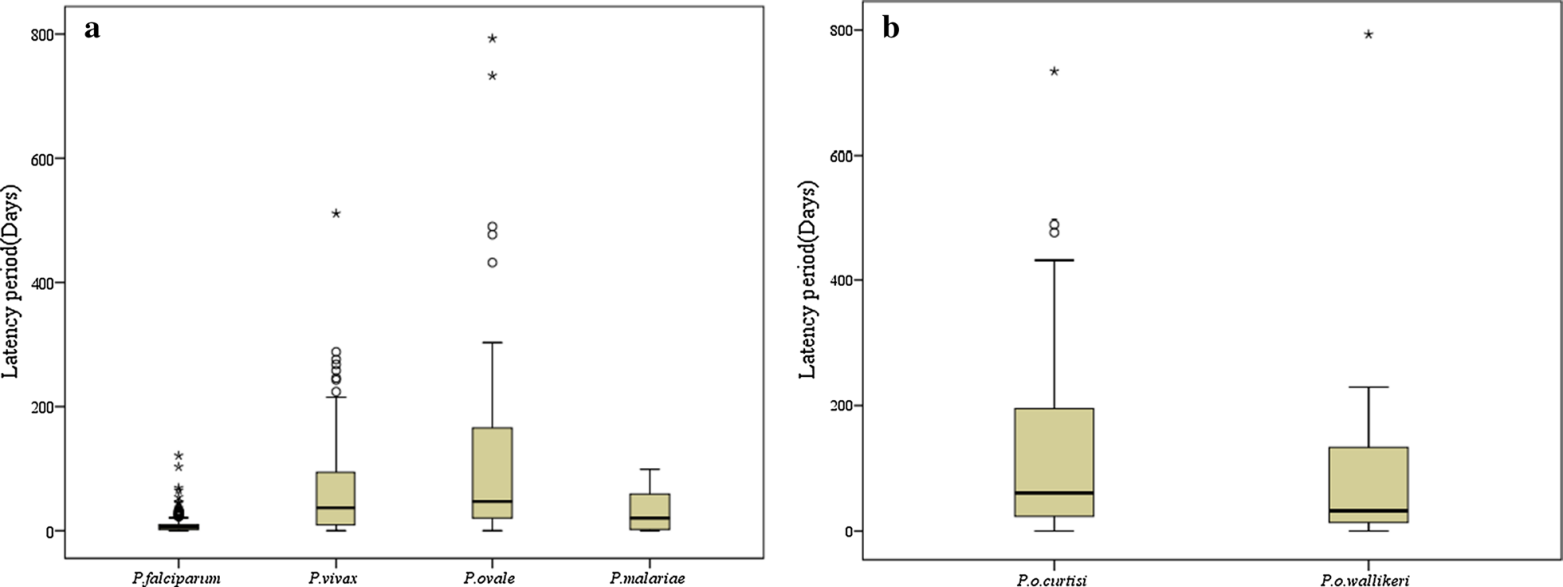
Latency periods for imported malaria cases in Hubei Province, 2014–2018. **a** Latency in days of cases caused by four *Plasmodium* species. **b** Latency in days of *P. ovale curtisi and P. ovale wallikeri* cases. The box plots show the latency period between arrival in China and illness onset. The midline of each box plot is the median, with the edges of the box representing the interquartile interval. Whiskers delineate the 5th and 95th percentiles. The black circles represent outliers of latency days. The asterisk represents the extreme of days elapsed between arrival in China and illness onset

The latency periods from returning to the country to the onset of symptoms are shown in Fig. 2. The median latency period (interquartile range, IQR) of *P. ovale* spp. was 47 (20.5–165.5) days, *P. malariae* was 20.5 (2.5–54.25) days, *P. vivax* was 37 (9.75–89) days, and *P. falciparum* was 6 (2–10) days. There was a significant difference in latency periods of four *Plasmodium* species (H = 176.93, p < 0.001). The median (IQR) latency periods of *P. o. curtisi* and *P. o. wallikeri* were 62 (24–195) days and 33 (14.25–129) days, respectively. There was no significant difference in the latency periods between *P. o. curtisi* and *P. o. wallikeri* (Z = − 1.308, p = 0.191).

### Interval from onset of illness to diagnosis

**Fig. 3:**
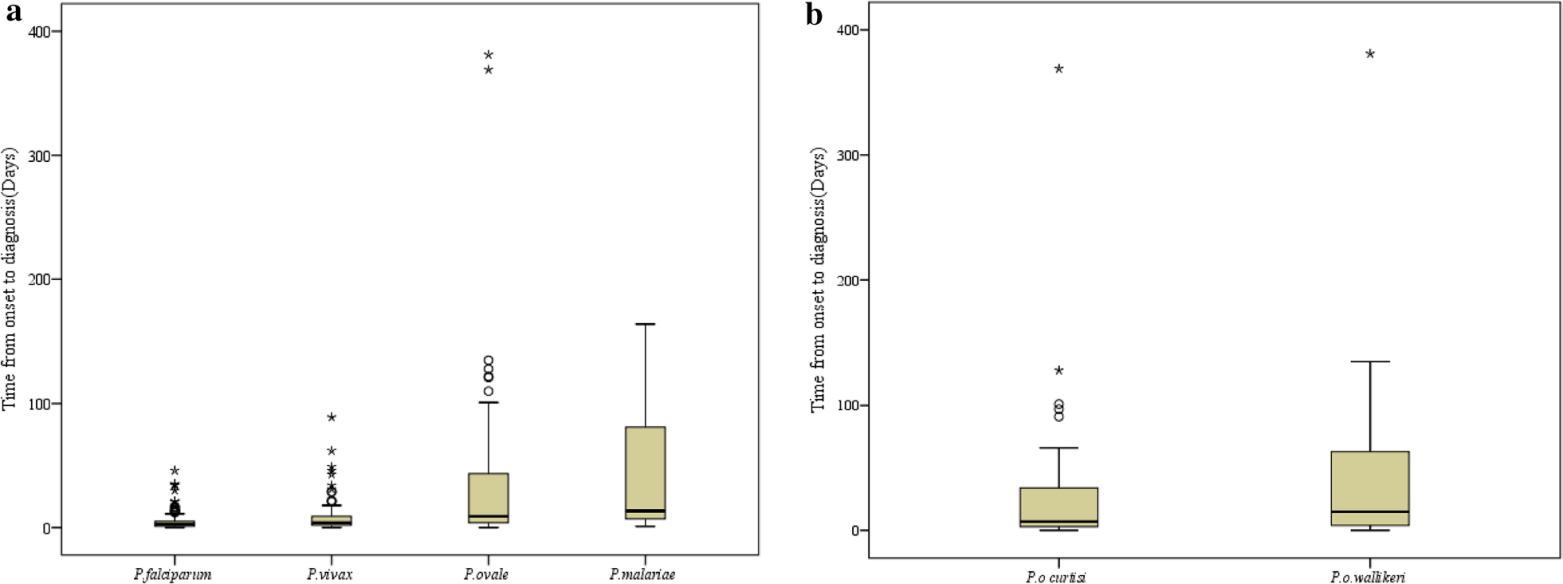
Interval from the onset of illness to diagnosis for imported malaria cases in Hubei Province, 2014–2018. **a** The interval from the onset of illness to diagnosis of malaria caused by four *Plasmodium* species. **b** The interval from the onset of illness to diagnosis of *P. ovale curtisi and P. ovale wallikeri* cases. The midline of each box plot is the median, with the edges of the box representing the interquartile interval. Whiskers delineate the 5th and 95th percentiles. The black circles represent the outliers of days elapsed between the interval from the onset of illness to diagnosis. The asterisk represents the extreme of days elapsed between the interval from the onset of illness to diagnosis

The intervals from onset of the illness to diagnosis are shown in Fig. 3. The median interval from the onset of illness to diagnosis (IQR) of *P. ovale* spp. was 9 (4–43.5) days, *P. malariae* was 13.5 (7.5–77.25) days, *P. vivax* was 4 (2–9) days, and *P. falciparum* was 3 (1–5) days. There was a significant difference in the interval from the onset of illness to diagnosis among four *Plasmodium* species (H = 48.168, p < 0.001). The median intervals from the onset of illness to diagnosis (IQR) of *P. o. curtisi* and *P. o. wallikeri* were 7 (3–34) days and 15 (4.25–62.75) days, respectively. There was no significant difference in the time from the onset of illness to diagnosis between *P. o. curtisi* and *P. o. wallikeri* (Z = 1.611, p = 0.107).

### Misdiagnosis of *P. ovale* spp. and *P. malariae* malaria cases

The misdiagnosis of *P. ovale* spp. and *P. malariae* is shown in Table 2. The initial malaria diagnosis was made via thick and thin blood film examination at hospitals or the lower-level CDC (county and city). Of 87 *P. ovale* spp. cases, only 42 had an accurate initial diagnosis, accounting for 48.3% of the *P. ovale* spp. cases. Similarly, of 18 *P. malariae* cases, only eight had an accurate initial diagnosis, accounting for 44.4% of all *P. malariae* cases.

**Table 2:** Misdiagnosis at initial identification in hospitals and low-level CDCs in Hubei

Confirmed diagnosis by PCR	Initial diagnosis					Total
	*P. ovale* spp.	*P. malariae*	*P. falciparum*	*P. vivax*	Mixed infection	
*P. ovale* spp.	42	3	3	38	1	87
*P. malariae*	0	8	1	9	0	18
Total	42	11	4	47	1	105

The sensitivity of microscopy and RDTs for diagnosing *P. ovale* spp. and *P. malariae* are shown in Table 3. In the Hubei Provincial Reference Laboratory for Malaria Diagnosis, 87 *P. ovale* spp. cases and 18 *P. malariae* cases were diagnosed by nested PCR. Of the 82 *P. ovale* spp. cases, 5 were misdiagnosed as *P. vivax* on microscopy. Of the 18 *P. malariae* cases, 2 were misdiagnosed as *P. vivax* on microscopy. RDT sensitivity of infections resulting from *P. ovale* spp. and *P. malariae* were 70.1% and 38.9%, respectively.

**Table 3:** Diagnosis of *P. ovale* ssp. and *P. malariae* malaria cases in Hubei Provincial Reference Laboratory for Malaria Diagnosis

	Confirmed diagnosis by PCR	
	*P. ovale* spp.	*P. malariae*
Microscopy result		
Correct diagnosis	82	16
Misdiagnosed	5	2
Sensitivity (%)	94.3	88.9
RDT result		
Positive^a^	61	7
Negative^b^	26	11
Sensitivity (%)	70.1	38.9

*RDT* Rapid diagnostic test

^a^ Indicating non-falciparum plasmodial infection

^b^ Indicating *Plasmodium* infection absent

### Origin of imported *P. ovale* spp. and *P. malariae* cases

**Fig. 4:**
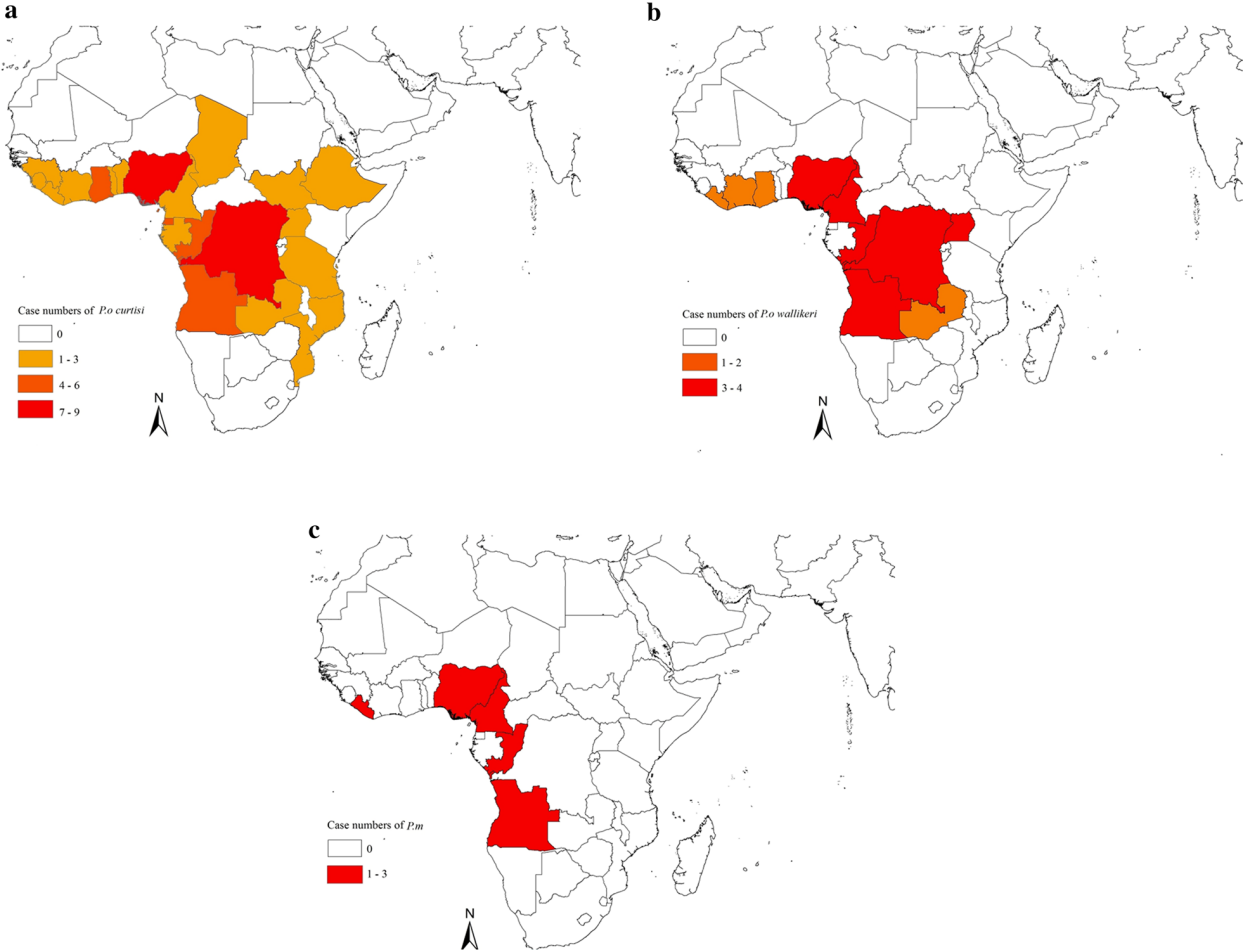
Geographic distribution of **a***Plasmodium ovale curtis*i; **b***Plasmodium ovale wallikeri;* and **c***Plasmodium malariae* originating from sub-Saharan Africa

During 2014–2018, the 87 imported *P. ovale* spp. cases and 18 imported *P. malariae* cases were acquired from 24 countries located in Africa, Asia, and Oceania. Overall, 97.7% (85/87) of *P. ovale* spp. cases and 94.4% (17/18) of *P. malariae* cases were from Africa, with only two *P. ovale* spp. cases from Asian countries and one *P. malariae* case from Oceania (Papua New Guinea). The countries of origin of cases imported from African countries are shown in Fig. 4.

98.4% (60/61) of *P. o. curtisi* and 96.2%(25/26) of *P. o. wallikeri* were from Africa. *P. o. curtisi* cases were mainly imported from Democratic Republic of Congo (9, 14.8%), Nigeria (7, 11.5%), Equatorial Guinea (6, 9.8%), Angola (5, 8.2%), and Congo (Brazzaville) (5, 8.2%), and one *P. o. curtisi* case from Turkey. *P. o.* wallikeri cases were mainly imported from Democratic Republic of Congo (4, 15.4%), Cameroon (4, 15.4%), Nigeria (4, 15.4%), Angola (3, 11.5%), Congo (Brazzaville) (3, 11.5%), and Uganda (3, 11.5%), and one *P. o. wallikeri* case from Bangladesh. *Plasmodium malariae* was mainly imported from Angola (3, 16.7%), Congo (Brazzaville) (3, 16. 7%), Cameroon (2, 11.1%), Liberia (2, 11.1%), and Nigeria (2, 11.1%).

## Discussion

Hubei Province has achieved remarkable success in controlling the local malaria situation; however, the number of imported malaria cases has increased. Although the majority of imported malaria cases were associated with *P. falciparum*, the number of patients with imported *P. ovale* spp. and *P. malariae* significantly increased, especially the number of patients with imported *P. ovale* spp. was more than that with *P. vivax* from 2014 to 2018. The increase in the number of imported *P. ovale* spp. and *P. malariae* cases presented a new challenge for achieving the long-term goal of malaria elimination [21].

In this study, there was a significant difference in the latency period of the four *Plasmodium* species. However, the difference between the two sympatric species of *P. ovale* spp. was not significant in this study, which is similar to the finding of Cao et al. [22] and Rojo-Marcos et al. [23]. In this study, the longest latency periods of *P. o. curtisi* and *P. o.e wallikeri* were 733 and 793 days, respectively. The longest latency periods of *P. o. curtisi* in the study by Nolder et al. [15] and Zhou et al. [24] were 1083 and 1265 days, respectively. Long latency periods present considerable difficulties for malaria diagnosis and its appropriate treatment [25]. The intervals from the onset of illness to diagnosis of *P. ovale* spp. and *P. malariae* were longer than those of *P. vivax* and *P. falciparum.* Because of the rare occurrence of indigenous *P. ovale* spp. and *P. malariae* malaria in Hubei Province, these *Plasmodium* species have been largely neglected. Moreover, some patients did not visit the hospital immediately because of their mild symptoms. However, delayed diagnosis of these two *Plasmodium* species will lead to worse clinical outcomes [26].

In this study, the rates of correct diagnosis of *P. ovale* spp. and *P. malariae* malaria cases were 48.3% and 44.4%, respectively, in hospitals or lower-level CDC. The misdiagnosis were driven by two factors, one was the health technicians’ lack of knowledge about these two species of malaria [27], and the other was morphological changes associated with low levels of parasitaemia and use of non-standard medications [28–31]. Therefore, training on using microscopy for initial diagnosis of malaria at hospitals and lower-level CDC should be strengthened. Periodic refresher training has helped maintain microscopy capabilities for accurate detection and diagnosis of *Plasmodium* parasites at all levels of CDC and hospitals; furthermore, a standard malaria testing procedure should be established at a lower level [32].

Identification of *Plasmodium* species is one of the important steps in malaria elimination. Common methods for the diagnosis of malaria include thick and thin blood film microscopy, RDTs, and PCR. The current gold standard for malaria diagnosis remains microscopy. However, this technique needs considerable training and experience. It was difficult to identify *P. ovale* spp. by only microscopic examination, because the morphology of this species is similar to that of *P. vivax* [29]. This study showed that five *P. ovale* spp. isolates were misidentified as *P. vivax* in microscopic examination in the provincial reference laboratory. PCR has higher sensitivity than microscopy in diagnosing *P. ovale* spp. and *P. malariae* infections [28, 33] which can be used to validate the laboratory diagnosis of *P. ovale* spp. and *P. malariae* infections [34]. RDTs provide access to accurate malaria diagnosis in many areas [35], because it is easy to use and require no specific equipment [36]. In this study, RDT sensitivity of infections resulting from *P. ovale* spp. was 70.1% which was suboptimal [37]. Hence, suggested that in hospitals or lower-level CDC, RDTs could be used to be a complementary part to microscopic examination of *P. ovale* spp. infections.

In this study, *P. ovale* spp. and *P. malariae* were acquired from sub-Saharan Africa. Collins and Jeffery [6] observed that the natural distribution of *P. ovale* spp. cases was in sub-Saharan Africa and some western Pacific islands. Both species of *P. ovale* spp. are considered to be sympatric throughout their geographical distribution [2]. However, *P. malariae* is mainly found in sub-Saharan Africa and southwest Pacific [7]. With the steadily increasing numbers of individuals working and travelling abroad to malaria-endemic Africa, malaria control in Hubei Province will likely remain an important challenge.

This study had some limitations. First, the database relied on passive case detection and inevitably could underestimate the numbers of imported malaria cases. Second, owing to the lack of an exact date of infection, the latency period was calculated using the arrival dates of patients when they returned to China, which means that the latency period of each case used in the study is a minimum estimate; the date of symptom onset relied on patient reporting, which is may be subject to recall bias and may therefore affect the confirmed diagnosis interval. Third, the small number of patients limited the statistical power to show differences among infections with different *Plasmodium* species.

## Conclusions

Imported malaria cases are the primary challenge for Hubei Province in achieving the long-term goal of malaria elimination. The study results demonstrated that the incidence of *P. ovale* spp. and *P. malariae* cases increased significantly. It also indicated the number of *P. ovale* spp. cases overtook that of *P. vivax* cases in Hubei Province. Nevertheless, these two species were most often misidentified or missed at hospitals or low-level CDC. Thus, suggested that more attention be paid to surveillance of imported *P. ovale* spp. and *P. malariae* cases to reduce the burden of public health and potential risk of malaria in China.

## Data Availability

The datasets used and/or analysed during the current study are available from the corresponding author on reasonable request.
